# Daily Inclusion of Resistant Starch-Containing Potatoes in a Dietary Guidelines for Americans Dietary Pattern Does Not Adversely Affect Cardiometabolic Risk or Intestinal Permeability in Adults with Metabolic Syndrome: A Randomized Controlled Trial

**DOI:** 10.3390/nu14081545

**Published:** 2022-04-08

**Authors:** Sisi Cao, Emily L. Shaw, William R. Quarles, Geoffrey Y. Sasaki, Priyankar Dey, Joanna K. Hodges, Avinash Pokala, Min Zeng, Richard S. Bruno

**Affiliations:** 1 Human Nutrition Program, The Ohio State University, Columbus, OH 43210, USA; cao.1142@osu.edu (S.C.); emsh95@gmail.com (E.L.S.); williamrquarles@gmail.com (W.R.Q.); sasaki.44@osu.edu (G.Y.S.); priyankar.dey@thapar.edu (P.D.); hodges.466@osu.edu (J.K.H.); pokala.2@osu.edu (A.P.); zeng.670@osu.edu (M.Z.); 2 Department of Biotechnology, Thapar Institute of Engineering and Technology, Patiala 147004, Punjab, India

**Keywords:** cardiometabolic health, metabolic syndrome, potato, endotoxin, intestinal permeability

## Abstract

Poor diet quality influences cardiometabolic risk. Although potatoes are suggested to adversely affect cardiometabolic health, controlled trials that can establish causality are limited. Consistent with potatoes being rich in micronutrients and resistant starch, we hypothesized that their inclusion in a Dietary Guidelines for Americans (DGA)-based dietary pattern would improve cardiometabolic and gut health in metabolic syndrome (MetS) persons. In a randomized cross-over trial, MetS persons (*n* = 27; 32.5 ± 1.3 year) consumed a DGA-based diet for 2 weeks containing potatoes (DGA + POTATO; 17.5 g/day resistant starch) or bagels (DGA + BAGEL; 0 g/day resistant starch) prior to completing oral glucose and gut permeability tests. Blood pressure, fasting glucose and insulin, and insulin resistance decreased (*p* < 0.05) from baseline regardless of treatment without any change in body mass. Oral glucose-induced changes in brachial artery flow-mediated dilation, nitric oxide homeostasis, and lipid peroxidation did not differ between treatment arms. Serum endotoxin AUC_0–120 min_ and urinary lactulose/mannitol, but not urinary sucralose/erythritol, were lower in DGA + POTATO. Fecal microbiome showed limited between-treatment differences, but the proportion of acetate was higher in DGA + POTATO. Thus, short-term consumption of a DGA-based diet decreases cardiometabolic risk, and the incorporation of resistant starch-containing potatoes into a healthy diet reduces small intestinal permeability and postprandial endotoxemia.

## 1. Introduction

Cardiovascular disease (CVD) is the leading cause of death among adults in the United States [1]. Because it requires decades to manifest, the evaluation of metabolic stressors throughout the sleep–wake–eating cycle has been increasingly emphasized because acute metabolic excursions precede overt CVD [2,3]. Indeed, an 8.8 year prospective trial (*n* = 22,514) showed that 2-h glucose concentrations following an oral glucose tolerance test is a better predictor of CVD-related mortality than fasting glucose [4]. The mechanism underlying this phenomenon is complex [2,3], but glucose excursions transiently impair vascular endothelial function (VEF) by increasing oxidative stress responses that limit the availability of nitric oxide to the vascular endothelium [3,5]. Poor gut health, including shifts in bacterial populations and intestinal permeability, also increases the translocation of inflammation-inducing endotoxins that initiate obesity and insulin resistance [6,7], which enhance CVD risk [8].

Poor diet quality and obesity are critical risk factors that influence a significant proportion of CVD cases [8]. The *Dietary Guidelines for Americans (DGA)* was developed based on evidence that foods, and, by extension, dietary patterns, can influence health and disease risk throughout the lifespan [9]. A key recommendation of the *DGA* is a dietary pattern rich in vegetables, but nearly 90% of Americans fail to meet goals for total vegetable intake [9]. Further, ~70–95% of Americans do not meet recommendations for each vegetable subcategory (dark green, red and orange, beans, peas, lentils, and starchy vegetables).

Despite the recognition that vegetables contribute to a healthy dietary pattern, controversy exists concerning the consumption of starchy vegetables, especially potatoes. Indeed, largely based on observational findings, potatoes may adversely affect cardiometabolic health [10,11,12,13]. In contrast, a systematic review [14] and evidence from prospective cohort studies [15] suggest that potatoes *per se* are unrelated to cardiometabolic risk (CVD, diabetes, and obesity). Rather, their adverse effects are likely attributed, at least in part, to how they are often prepared (e.g., French fries, and chipped) [14,16]. These opposing views support a need for randomized controlled trials to establish causality whether CVD risk is influenced by a dietary pattern containing potatoes.

Health concerns regarding potatoes are commonly associated with it being a high-glycemic food [17]. However, potatoes are nutrient-dense and provide dietary fiber and essential micronutrients, including high levels of potassium [13,17]. While conventionally prepared potatoes (e.g., boiled, and baked) that are immediately consumed exhibit a glycemic response similar to refined bread [18], potatoes that are cooked and, subsequently, cooled prior to ingestion have increased resistant starch content [19]. Resistant starch withstands enzymatic hydrolysis in the small intestine and reaches the colon where it can promote prebiotic activity [19,20]. Indeed, supplementation of potato starch (3 d; 48 g) in humans decreased Firmicutes: Bacteroidetes and increased fecal short-chain fatty acids (SCFAs) [21]. This suggests that RS-containing potatoes may favorably regulate gut barrier function and glucose excursions that otherwise impair VEF.

No randomized controlled studies have examined whether resistant starch-containing potatoes can be incorporated effectively into a *DGA*-based dietary pattern to improve cardiometabolic health. We hypothesized that consumption of resistant starch-containing potatoes as part of a *DGA*-based dietary pattern would improve gut barrier function and attenuate postprandial hyperglycemia that otherwise impairs VEF. To test this, we conducted a randomized controlled crossover trial in persons with metabolic syndrome (MetS) because of their heightened CVD risk [8] that often occurs in association with glucose intolerance, impaired VEF [22], gut dysbiosis, and intestinal barrier dysfunction [23]. MetS persons were provided eucaloric *DGA* diets for 2 weeks that contained resistant starch-containing potatoes (DGA + POTATO) or a bagel (DGA + BAGEL). Assessments of VEF (primary outcome) and nitric oxide homeostasis, endotoxin (secondary outcome) and gut barrier functions, and cardiometabolic risk factors were then performed at fasting and/or during a 2-h postprandial period following an oral glucose tolerance test. Outcomes of this controlled study are therefore expected to help clarify cardiometabolic risks affected by potatoes while also informing the design of longer-term studies.

## 2. Materials and Methods

### 2.1. Participants

The study protocol was approved by the Institutional Review Board at The Ohio State University (2018H0265) and registered at ClinicalTrials.gov (NCT03624569). All participants were recruited from the Columbus, Ohio, area and all aspects of the intervention were performed at the OSU Metabolic Health Laboratory from August 2018 through to July 2019. Written informed consent was obtained from each subject prior to performing any study procedures.

Men and women between 18–50 years of age who met gender-specific criteria for MetS were eligible to participate in this study. Prior to enrollment, height, weight, waist circumference, and resting blood pressure were measured, and a fasting blood sample was obtained. Waist circumference was determined at the level of the umbilicus and blood pressure was reported as the mean of two measurements taken 1 min apart. MetS was defined by the presence of ≥3 of 5 diagnostic criteria [24]: glucose (≥100 mg/dL); triglyceride (≥150 mg/dL); waist circumference ≥ 88 cm for women or ≥102 cm for men; HDL-C < 50 mg/dL for women or <40 mg/dL for men; and resting blood pressure ≥ 130/85 mmHg. Participants were also required to meet the following inclusion criteria: weight stable (±2 kg during past 3 month); nonsmoker; non-user of dietary supplements including probiotics during past 1 month; no use of medications known to affect glucose, lipids, or blood pressure; consumption of <3 alcoholic beverages daily; participation in <7 h/week of aerobic activity; no history of gastrointestinal disorders or food allergies or intolerance. Women were also required to be pre-menopausal, non-users of oral contraceptives or no change in birth control during the prior 3 month, and not pregnant or lactating.

### 2.2. Study Design

In this randomized controlled, crossover trial, participants with MetS completed two 14-d study interventions separated by a 2-week washout period. This timeline permitted women to complete each study intervention during the same phase of their menstrual cycle to control the confounding effects of sex hormones on FMD responses [25]. For each intervention period, all foods were provided to participants in a eucaloric manner based on their estimated energy requirements, which were calculated using the Harris-Benedict equation [26]. Diets closely mimicking *DGA* recommendations were provided to participants on a 4-day rotating basis [9]. Participants’ prescribed diet quality was confirmed by calculating the Healthy Eating Index (HEI) score, an index that was developed as a composite metric of diet quality that aligns with the key *DGA* recommendations [27]. Prescribed meal patterns had scores of approximately 98 out of a possible 100 points. Supplemental Table S1 shows the nutrient composition, daily servings of food groups, and HEI scores of the 2500 kcal diet during the DGA + POTATO and DGA + BAGEL study arms. Prescribed foods were identical between study arms except for the daily inclusion of a potato (350 g, Russet Burbank) or a bagel (100 g, Lender’s plain; Table 1). However, serving sizes of select foods required minor adjustments to closely match the diets for each food group and HEI scores.

During each intervention, participants visited the study center on days 0, 7, and 14 for anthropometric and blood pressure measures. Fasting blood samples were also collected on days 0 and 14. On day 14, participants provided a fecal sample that was self-collected during the preceding 24 h. From an in-dwelling catheter placed in the antecubital vein, a fasting blood sample was collected and ultrasound-based measures of vascular health, specifically brachial artery flow-mediated dilation (FMD), and carotid artery intima-media thickness (cIMT), were obtained (described below). Thereafter, participants ingested a 240 mL 75 g oral glucose tolerance beverage (Thermo Fisher Scientific, Waltham, MA, USA, #401025P). This beverage also contained non-digestible sweeteners (sucralose, erythritol, mannitol, and lactulose) that were used to assess intestinal permeability based on their 24-h urinary excretion (described below). In addition to the fasted blood sample (0 min), blood samples were collected at 30, 60, 90, and 120 min to assess metabolic excursions during the 2-h postprandial period. Brachial artery FMD was also assessed at these same time points. Following the intervention, participants completed a 2-week washout before returning to the study center to again complete the study as described, but with allocation to the alternative treatment.

### 2.3. Dietary Control, Assessment, and Compliance

Participants were provided identical foods for each 2-week intervention period in a eucaloric manner to maintain body mass. Consistent with the *DGA* [9], the daily target was 45–55% of total energy from carbohydrate, <30% from fat, and 15–20% from protein. Saturated fat was restricted to <10% of total energy, sodium to <2300 mg/day, and dietary fiber was provided at 15 g/1000 kcal. To standardize the resistant starch content and promote compliance, potatoes were microwaved and prepared plain; mashed with oat milk and a butter alternative; cut into wedges and lightly seasoned with sodium-free spices; or sliced and topped with dairy-free cheese and soy milk (au gratin). Regardless of preparation style, all cooked potatoes were refrigerated for 24–72 h prior to consumption to promote retrogradation and enhanced resistant starch content (described below). DGA + BAGEL provided ~4 g/day of resistant starch in agreement with Americans’ usual dietary patterns [29] whereas the resistant starch content of DGA + POTATO was ~20 g/day with 17.5 g derived from the retrograded potato (Table 1). Bagel and potato resistant starch content were determined using a commercially available spectrophotometric kit based on AOAC 2002.02 methodology according to the manufacturer’s instructions (Megazyme, Wicklow, Ireland; #K-RSTAR).

To assess participants’ dietary compliance, all food containers were returned to the study center to weigh any uneaten food items. In addition, participants maintained a daily food log to document any deviations from the prescribed diet. Actual dietary energy and nutrient intakes were assessed using the Nutrition Data System for Research software version 2018 (University of Minnesota, Minneapolis, MN, USA) from weighed food records and with consideration of the ingestion of any non-prescribed foods. Dietary compliance was pre-defined based on two complementary metrics: (1) >80% consumption of the bagel or potato in both study arms (per cent of total food weight consumed), and (2) achieving a mean HEI score of >90 for each 2-week intervention.

### 2.4. Biospecimen Collection and Processing

Whole blood was collected into evacuated blood tubes containing no anticoagulants to prepare serum or into EDTA- or sodium heparin-containing tubes to prepare plasma. Serum or plasma was obtained by centrifugation (3000× *g*, 4 °C, 15 min), aliquoted, and snap-frozen in liquid nitrogen prior to storing at −80 °C until analyzed. At the time of sample processing, an aliquot of sodium heparinized plasma was mixed 1:1 with 10% perchloric acid containing 1 mM diethylenetriaminepentaacetic acid, centrifuged (15,000× *g*, 4 °C, 5 min), and the supernatant was snap-frozen and stored at −80 °C for future analysis of ascorbic acid and uric acid.

During each 14-day trial, participants were instructed to collect their fecal samples within 24 h of their scheduled visit to the study center on day 14. Fecal samples were maintained under refrigerated conditions using ice packs until samples were provided to study personnel. Samples were then categorized using a 7-point Bristol Stool Scale [30], aliquoted, and stored at −80 °C. Complete urine samples were also collected for 24 h on day 14 through day 15 after participants ingested the oral glucose tolerance beverage that also contained non-digestible sugar probes. Participants received separate collection containers to collect urine from 0–5 h and 6–24 h to assess gastrointestinal permeability (described below).

### 2.5. Clinical Chemistries, Metabolic Hormones, and Endotoxemia

Plasma glucose (G75171L), triglyceride (T7532500), and HDL-cholesterol (H751160) were measured using separate commercially available clinical assays (Pointe Scientific; Canton, MI, USA) on a UV-2600 tabletop spectrophotometer (Shimadzu). Plasma cholecystokinin (LifeSpan Biosciences, Seattle, WA, USA; LS-F5655) and insulin (Alpco, Salem, NH, USA; 80-INSHU-E10.1) were measured by ELISA following the manufacturer’s instructions using a Synergy H1 microplate reader (Biotek Instruments, Winooski, VT, USA). The homeostatic model assessment of insulin resistance (HOMA-IR) was calculated using fasting plasma glucose and insulin as described [31]: ((glucose (mmol/L) × insulin (µIU/mL)/22.5).

Serum endotoxin was measured using a high-sensitivity recombinant factor-C (rFC)-based fluorometric assay using endotoxin-free supplies (Lonza, Basel, Switzerland; PyroGene rFC 50-658U) as we described [32]. In brief, serum was diluted in endotoxin-free water, incubated (70 °C, 10 min), and mixed with the supplied reagent containing rFC prior to measuring fluorescence before and after 1-h incubation. The change in sample fluorescence was then used to calculate serum endotoxin concentration using an endotoxin standard curve that was prepared in parallel.

### 2.6. Vascular Function

cIMT was assessed on day 14 of each trial, using a Terason T3000 ultrasound system as described [33]. Because cIMT values did not differ between trials (*p* > 0.05), mean values are reported. Using the same ultrasound system, brachial artery FMD was assessed as we described [5,22,34] on day 14 from 0 min through 120 min at 30-min intervals. All measures and analyses of images were performed by the same technician who was blinded to study treatment. In brief, the transducer was placed above the antecubital crease of the right arm for imaging. Pre-occlusion diameter of the brachial artery was recorded for 1 min. An automated blood pressure cuff placed distal to the olecranon process was then inflated (200 mmHg) to occlude blood flow for 5 min. Post-occlusion vessel diameters recordings were monitored for 1 min prior to and 3 min following rapid cuff deflation. Following image analysis with edge-detection software (Medical Imaging Applications), brachial artery FMD (%) was calculated as: (peak post-occlusion diameter (mm) − pre-occlusion diameter (mm))/pre-occlusion diameter (mm) × 100. Shear rate area under the curve (AUC) was calculated from the time of cuff release to peak post-occlusion diameter after initially calculating shear rate as described [25]: 4 × ((mean blood velocity; m/s)/end-diastolic diameter (mm)).

### 2.7. Arginine Metabolism and Nitric Oxide Metabolites

Plasma arginine (ARG) and its vasoactive metabolites asymmetric dimethylarginine (ADMA), symmetric arginine (SDMA), and homoarginine (homoARG) were measured by HPLC-FL as we detailed [5]. Plasma nitrate and nitrite (NO_x_), the end-product metabolites of nitric oxide, were assessed to estimate nitric oxide status using a kit (Cayman Chemical; 780001). Measures were performed according to the manufacturer’s instructions to include an initial filtration of plasma using a 30 kDa molecular weight cut-off filter (Amicon, Burlington, MA, USA; UFC500396).

### 2.8. Lipid Peroxidation and Plasma Antioxidants

The lipid peroxidation biomarker malondialdehyde was measured from plasma by HPLC-FL following derivatization with thiobarbituric acid and extraction with butanol as we described [5]. Plasma ascorbic acid and uric acid were by HPLC-electrochemical detection from perchloric acid-treated plasma as we described [5], with minor modification to use a Thermo Dionex Ultimate 3000 HPLC system equipped with two 6011RS ultra electrochemical cells set to oxidation potentials of 50 mV, 250 mV, 350 mV, and 450 mV.

### 2.9. Gastrointestinal Permeability Test

On day 14, participants ingested 1 g sucralose (Spectrum, #S1416), 1 g erythritol (Spectrum, New Brunswick, NJ, USA, #E1494), 1 g mannitol (Thermo Fisher Scientific, Waltham, MA, USA, #18-604-224), and 5 g lactulose (Akorn Pharmaceuticals, Lake Forest, IL, USA, #50383-795-16) concurrent with the oral glucose tolerance test. From complete 24-h urine samples collected from 0–5 h and 6–24 h, upper and lower gastrointestinal permeability was assessed by LC-MS [35]. During this 24-h period, all meals were provided and devoid of the above sweeteners. In brief, urine samples were diluted in acetonitrile:water (85:15), centrifuged (10,000× *g* 15 min, 4 °C), and the supernatant was injected on a Prominence LCMS-2020 instrument (Shimadzu). Isocratic separation was performed at 0.2 mL/min on an Acquity UPLC BEH Amide column (100 × 2.1 mm; 1.7 μm; Waters Corp) using acetonitrile:water (65:35) containing 0.1% (*v*/*v*) ammonium hydroxide as the mobile phase. Single-ion monitoring following negative mode electrospray ionization was used to detect each compound at their mass/charge ratios at their corresponding retention times against authentic standards. Area ratios of authentic standards against appropriate internal standards (^13^C glucose for sucralose and erythritol; ^13^C-mannitol for mannitol, ^13^C-sucrose for lactulose) were used to quantify each sugar probe. Excretion ratios of lactulose/mannitol and sucralose/erythritol at 0–5 h and 6–24 h were calculated to assess proximal and distal intestinal permeability, respectively, as described [36,37].

### 2.10. Gut Microbiome and Fecal Short-Chain Fatty Acids

Fecal samples collected within 24 h of the day 14 study visit were used to assess gut microbial community structure. Bacterial DNA was extracted using a QIAamp Fast DNA Stool Mini Kit (Qiagen, Hilden, Germany) as described [38]. Then, the V4–V5 hypervariable region of the 16S rRNA gene was amplified by PCR to prepare amplicon libraries followed by MiSeq sequencing (Illumina, San Diego, CA, USA) [39]. Paired-end reads (2 × 300 paired-end protocol) were analyzed using QIIME2 (version 2020.2) [40]. The initial workflow involved removing primers and spacers from sequences and the trimmed sequences were then processed by DADA2 to denoise, merge forward and reverse reads, and remove chimeric sequences [41]. Forward and reverse reads were trimmed when the quality score of reads was <30. A rarefied feature table was then used to assess α- (e.g., phylogenic diversity, Chao1, Shannon) and β-diversity (e.g., Bray-Curtis dissimilarity). A Naïve Bayes classifier using the 99% 16S reference reads data set and raw.taxonomy files from the Silva database (release 132) were used to create a trained classifier. The trained classifier was then applied to the DADA2 feature table to assign the appropriate taxonomies.

A panel of 9 straight- and branched-chain SCFAs was also assessed from fecal samples as described [42], with minor modifications. In brief, samples were derivatized with 3-nitrophenylhydrazine (3NPH) to convert each SCFA to a corresponding 3-nitrophenylhydrazone. Samples were separated on an Acquity UPLC BEH C18 column (2.1 × 100 mm, 1.7 μm; Waters Corp, Milford, MA, USA) using a binary gradient as described [42], and 3NPH-SCFAs were detected at their corresponding mass-to-charge ratios using a Shimadzu LCMS-2020 system following electrospray ionization (negative mode) and single ion monitoring. Mass spectra were acquired with an interface voltage of 4.5 kV, a detector voltage of 1.55 kV, a heat block temperature of 400 °C, and a desolvation gas temperature of 250 °C. Nitrogen was used as the nebulizer gas (1.5 L/min) and drying gas (15 L/min). Quantification was performed using external standards prepared in parallel and against ^13^C_4_-butyrate (internal standard; Cambridge Isotopes, Tewksbury, MA, USA).

### 2.11. Statistical Analyses

Fasting serum endotoxin and brachial artery FMD AUC_0–2 h_ were the pre-defined primary and secondary outcomes of the study. Study powering was initially based on an estimated 25% decrease in endotoxin using data showing that endotoxin in MetS persons is approximately double those of healthy persons [43]. A separate analysis was then performed using brachial artery FMD responses obtained in MetS persons during a 3-h postprandial period following a high-carbohydrate meal challenge [22]; a 25% attenuation in FMD responses was predicted. Our analyses indicated that 26 participants would be needed to achieve 80% study powering (*p* < 0.05). Therefore, we aimed to enroll 30 persons to account for potential attrition and non-compliance.

Data (means ± SE) were analyzed using R statistical analysis software (version 4.0.3). Baseline characteristics were analyzed between men and women with MetS using a Student’s *t*-test for ordinal data or the Fisher’s exact test for categorical data. Any effects due to time (day 0 vs. day 14), treatment (bagel vs. potato), and time × treatment interactions were determined using 2-way repeated measures ANOVA with Bonferroni’s post-test. Prior to statistical testing, multiple imputation using predictive mean matching [44] was applied to account for any missing values in the dataset. Five simulations of missing data were performed, and separate analyses with 2-way repeated measures ANOVA were conducted prior to parameter pooling according to Rubin’s rules [45]. To evaluate between-treatment effects during the 2-h postprandial period, the AUC responses of each variable for each subject were calculated using the trapezoidal rule. Any measures performed exclusively on day 14 (e.g., SCFAs, and gut permeability probes) were analyzed using a paired *t*-test to assess between-treatment effects. For gut microbiome, differential abundance analysis for between-treatment effects was conducted using ANCOM-II, which accounts for repeated measures, has high power, and applies a low-threshold for false discovery rate [46,47]. ANCOM II was implemented with R software using published source code [48]. Data were preprocessed using the following parameters: group_var = NULL, out_cut = 0.5, zero_cut = 0.90, lib_cut = 0, neg_lb = FALSE). Analysis was performed on the processed feature table using default parameters. Multiple comparisons were accounted for using the Benjamini–Hochberg method. Statistical significance for all analyses was set at *p* ≤ 0.05.

## 3. Results

### 3.1. Participants, Compliance, & Dietary Intakes

**Figure 1:**
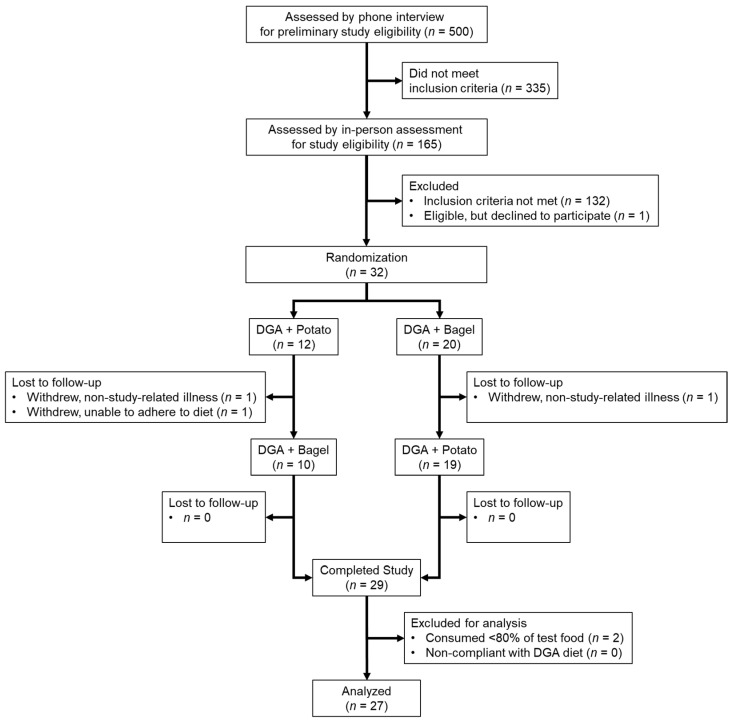
Enrollment and follow-up of study participants with MetS. Participants completed a randomized crossover study to examine daily potato (DGA + POTATO) or bagel (DGA + BAGEL) while following a prescribed *DGA* diet. MetS persons completed the study with no adverse events, but 3 were lost to follow-up and 2 were non-compliant to study procedures. Abbreviations: *DGA*, *Dietary Guidelines for Americans*; and MetS, metabolic syndrome.

A total of 32 persons with MetS were randomized for study entry (Figure 1). Of these, 3 were lost during follow-up due to unrelated illness or an inability to consume the prescribed diet. The remaining 29 participants completed both study arms without any adverse effects, but 2 were excluded from the final analysis because they consumed 51–55% of the test food, which was well below the predefined level of 80% for compliance. Thus, the final analysis included 27 persons with MetS (*n* = 13 men and 14 women). These persons consumed > 94% of the prescribed bagels or potatoes and had near-maximal mean HEI scores that did not differ between each 14-day intervention (DGA + BAGEL, 98.1 ± 0.2 vs. DGA + POTATO, 98.0 ± 0.1; *p* > 0.05), suggesting excellent compliance to the prescribed diets regardless of study phase.

On average, participants fulfilled MetS criteria based on elevated glucose, low HDL-C, and increased waist circumference (Table 2). Men had higher circulating triglyceride and systolic blood pressure than women. Men and women did not differ in the proportion of participants meeting 3, 4, or 5 MetS risk factors. Further, a similar proportion of men and women had increased waist circumference and blood pressure, and depressed HDL-C; fewer men than women had elevated fasting glucose; and more men had hypertriglyceridemia. Participants’ plasma ascorbic acid and uric acid concentrations were within normal limits [49], and men had higher uric acid than women consistent with known sex differences [49]. Lastly, all participants, regardless of gender, were at low risk for atherosclerosis based on established criteria for left and right cIMT [33].

Plasma ascorbic acid and uric acid were evaluated only on day 14. While there was no statistically significant difference in ascorbic acid concentrations on day 14 between DGA + BAGEL and DGA + POTATO (47.5 ± 2.9 vs. 52.5 ± 4.8 μmol/L), ascorbic acid concentrations on day 14 in DGA + POTATO but not DGA + BAGEL were increased compared to those measured at participant screening (~2–3 weeks before day 0; 40.9 ± 4.2 μmol/L). Uric acid concentrations on day 14 did not differ between study arms nor did they differ from concentrations at screening (data not shown).

### 3.2. Changes in Cardiometabolic Health Parameters following the 2-week Intervention

Our first approach to test our hypothesis was to consider whether the 2-week intervention could favorably improve cardiometabolic health in MetS persons on day 14 relative to day 0. Consistent with providing prescribed eucaloric diets, participants’ BMI and waist circumference were unaffected (*p* > 0.05; Table 3) by time (day 0 vs. 14) or treatment (bagel vs. potato). Systolic and diastolic blood pressure did not differ between DGA + BAGEL and DGA + POTATO, but both systolic and diastolic blood pressure decreased in response to the DGA-based diets (P_Time_ < 0.001). Statistically significant decreases over time were also observed for fasting glucose, insulin, and HOMA-IR (Table 3). We also assessed several other cardiometabolic biomarkers at fasting on day 0 and day 14 of each study arm. No statistically significant effects due to time, treatment, or time × treatment interactions were observed for circulating concentrations of cholecystokinin, NO_x_, ARG, or ADMA (Table 3). However, SDMA but not SDMA/ARG increased due to time (*p* = 0.04) regardless of treatment. homoARG but not homoARG/ARG also tended to increase due to time and treatment (*p* = 0.07–0.10). These findings suggest that a short-term intervention based on a *DGA* dietary pattern can favorably improve select aspects of cardiometabolic health, especially glycemia, insulinemia, and blood pressure, without any influence from daily potato consumption.

### 3.3. Intervention Effects on Fasting Endotoxemia and Vascular Health

Metabolic endotoxemia, based on circulating endotoxin, and brachial artery FMD as an index of VEF were the pre-defined primary and secondary outcomes, respectively, of this randomized controlled trial. Although we hypothesized that endotoxemia and VEF would improve in response to DGA + POTATO, neither serum endotoxin (*p* > 0.05; Table 3) nor brachial artery FMD (DGA + BAGEL vs. DGA + POTATO, 7.2 ± 1.3% vs. 8.7 ± 1.3%; *p* = 0.40) were significantly different between study arms at fasting on day 14. Similarly, pre-occlusion diameter (4.22 ± 0.11 mm vs. 4.03 ± 0.12 mm; *p* = 0.24) and post-occlusion diameter (4.51 ± 0.11 mm vs. 4.37 ± 0.12 mm; *p* = 0.41) of the brachial artery were not different between DGA + BAGEL and DGA + POTATO. However, the post-occlusion brachial artery diameter was increased compared with the pre-occlusion diameters in both the DGA + BAGEL (4.51 ± 0.11 mm vs. 4.22 ± 0.11 mm) and DGA + POTATO study arms (4.37 mm ± 0.12 vs. 4.03 ± 0.12 mm; *p* < 0.0001). Increases in post-occlusion relative to pre-occlusion artery diameters also occurred in the absence of an occlusion period × treatment interaction (*p* = 0.38) or significant time, treatment, or interactive effects on arterial shear stress (*p* > 0.05; data not shown) to support the concept that a *DGA*-based dietary pattern can improve vascular health.

### 3.4. Intervention Effects on Postprandial Glycemia, Cholecystokinin, and Lipid Peroxidation

**Figure 2:**
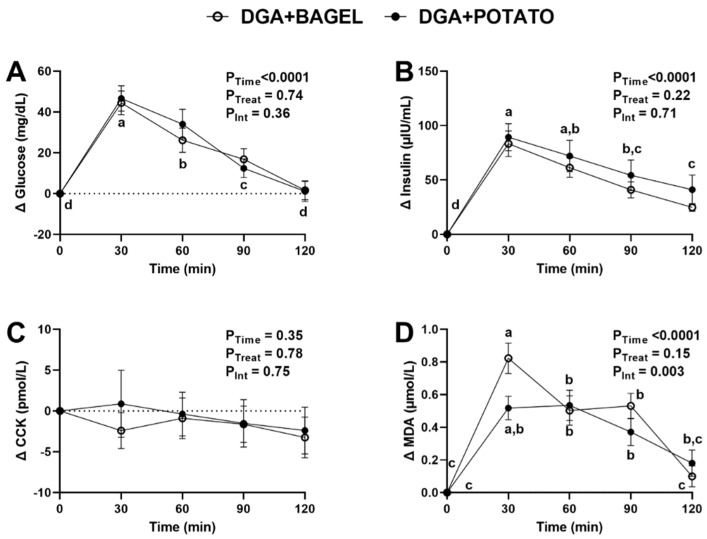
Plasma metabolic excursions in MetS persons who followed a *DGA*-based diet containing potatoes (DGA + POTATO) or bagels (DGA + BAGEL) for 2 weeks prior to completing a glucose tolerance test. (**A**) Plasma glucose, (**B**) insulin, (**C**) CCK, and (**D**) MDA during a 2-h postprandial period following the ingestion of 75 g of glucose. Data (means ± SE, *n* = 27) are expressed as changes from baseline (0 min) and were analyzed by 2-way RM ANOVA with Bonferroni’s post-test to assess effects due to time, treatment, and their interaction. *p* < 0.05 was considered statistically significant, and data are annotated (a > b > c) to visualize post-hoc effects due to time or time x treatment as appropriate. Abbreviations: CCK, cholecystokinin; *DGA*, *Dietary Guidelines for Americans*; MDA, malondialdehyde; and MetS, metabolic syndrome.

Consistent with evidence that acute hyperglycemia increases CVD risk [2,3], the main focus of the intervention was to examine postprandial changes in cardiometabolic risk factors in response to an oral glucose challenge. As expected, the oral glucose challenge increased plasma glucose by 30 min and subsequently decreased in a time-dependent manner (Figure 2A). No treatment or time × treatment interaction was detected. A similar time-dependent change for postprandial insulin was observed in response to the glucose challenge (Figure 2B) but postprandial cholecystokinin exhibited no effects due to time, treatment, or their interaction (Figure 2C). Postprandial malondialdehyde exhibited a significant time × treatment interaction (*p* = 0.003; Figure 2D) suggesting that time-dependent responses differed between treatments. Post-hoc analysis indicated that malondialdehyde increased by 30 min regardless of treatment. Although malondialdehyde returned to concentrations no different from fasting concentrations regardless of treatment, its concentrations at 120 min in DGA + POTATO were significantly lower than those at 90 min whereas no difference in concentration was observed at 90 min vs. 120 min for DGA + BAGEL. To fully consider potential between-treatment effects, we also calculated plasma AUC_0–120 min_ for each variable (Table 4). AUC_0–120 min_ for glucose, insulin, and cholecystokinin did not differ between DGA + BAGEL and DGA + POTATO, but malondialdehyde AUC_0–120 min_ tended (*p* = 0.08) to be lower in DGA + POTATO compared with DGA + BAGEL (Table 4).

### 3.5. Intervention Effects on Postprandial Vascular Health and Nitric Oxide Homeostasis

**Figure 3:**
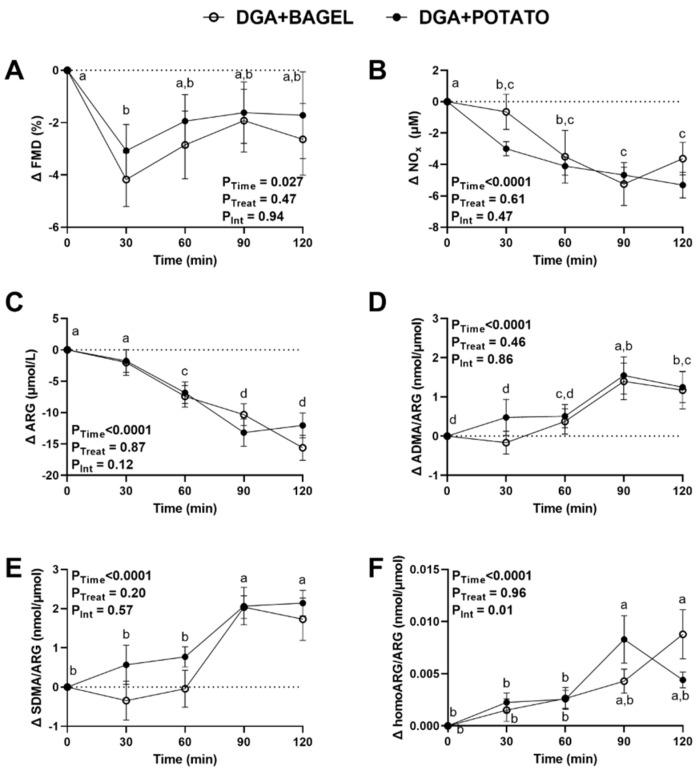
Vascular endothelial function and plasma biomarkers of nitric oxide homeostasis in MetS persons who followed a *DGA*-based diet containing potatoes (DGA + POTATO) or bagels (DGA + BAGEL) for 2 w prior to completing an oral glucose tolerance test. (**A**) Brachial artery FMD, and plasma (**B**) NOx, (**C**) ARG, (**D**) ADMA/ARG, (**E**) SDMA/ARG, and (**F**) homoARG/ARG during a 2-h postprandial period following the ingestion of 75 g of glucose. Data (means ± SE, *n* = 27) are expressed as changes from baseline (0 min) and were analyzed by 2-way RM ANOVA with Bonferroni’s post-test to assess effects due to time, treatment, and their interaction. *p* < 0.05 was considered statistically significant, and data are annotated (a > b > c) to visualize post-hoc effects due to time or time × treatment as appropriate. Abbreviations: ADMA, asymmetric dimethylarginine; ARG, arginine; *DGA*, *Dietary Guidelines for Americans*; FMD, flow-mediated dilation; homoARG, homoarginine; MetS, metabolic syndrome; NOx, nitric oxide metabolites (sum of nitrate and nitrite); and SDMA, symmetric dimethylarginine.

Following the oral glucose challenge, brachial artery FMD responses decreased at 30 min and returned to levels no different from 0 min by 60 min regardless of study arm (P_Time_ = 0.027; Figure 3A). The lack of treatment effect on FMD responses was corroborated by examining the between-trial FMD AUC_0–120 min_, which showed no statistically significant effect (Table 4). Postprandial plasma concentrations of NO_x_ and ARG exhibited statistically significant main effects for time (*p* < 0.0001; Figure 3B,C), with both decreasing and without normalization to baseline concentrations. Both ADMA/ARG and SDMA/ARG increased with time regardless of treatment (*p* < 0.0001; Figure 3D,E). ADMA/ARG and SDMA/ARG were both increased at 90–120 min compared to 0–30 min. homoARG/ARG exhibited a time × treatment interaction (Figure 3F). Post-hoc analysis indicated that homoARG/ARG in DGA + POTATO increased at 90 min before returning to levels at 120 min that were not different from baseline. In contrast, homoARG/ARG in DGA + BAGEL increased at 120 min, which did not significantly differ from time-matched levels in DGA + POTATO. Lastly, between-trial comparisons of AUC_0–120_ min for FMD, NOx, ARG, ADMA/ARG, SDMA/ARG, and homoARG/ARG showed no between-trial differences (*p* = 0.11–0.98; Table 4) consistent with our lack of detection of any treatment effects for any of these study variables (Figure 3). These findings suggest that, while an oral glucose challenge impairs vascular health and nitric oxide homeostasis, the daily inclusion of potatoes as part of a *DGA* dietary pattern does not improve or adversely affect these parameters of cardiometabolic health.

### 3.6. Intervention Effects on Postprandial Endotoxemia and Gut Permeability

**Figure 4:**
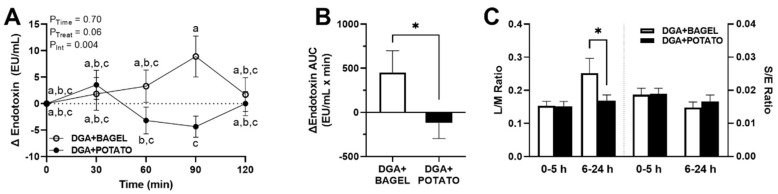
Postprandial endotoxemia and gut permeability in MetS persons who followed a *DGA*-based diet containing potatoes (DGA + POTATO) or bagels (DGA + BAGEL) for 2 w prior to completing simultaneous oral glucose tolerance and gut permeability tests. (**A**) Serum endotoxin and (**B**) endotoxin AUC_0–120 min_ during a 2-h postprandial period and (**C**) 24-h urinary L/M and S/E following the ingestion of 75 g of glucose and non-digestible sugar probes. Serum endotoxin (means ± SE, *n* = 27) are expressed as change from baseline (0 min) and were analyzed by 2-way RM ANOVA with Bonferroni’s post-test to assess effects due to time, treatment, and their interaction. *p* < 0.05 was considered statistically significant, and data are annotated (a > b > c) to visualize post-hoc effects due to time or time x treatment as appropriate. Endotoxin AUC_0–120 min_ was calculated using the trapezoidal rule. Endotoxin AUC_0–120 min_ and time-dependent analyses of L/M and SE were analyzed using a paired Student’s *t*-test (* *p* < 0.05). Abbreviations: AUC, area under the concentration curve; *DGA*, *Dietary Guidelines for Americans*; L/M, lactulose/mannitol; and S/E, sucralose/erythritol.

On day 14 of each study arm, in addition to the oral glucose challenge, participants concurrently ingested non-digestible sugar probes to test the hypothesis that the potato-based dietary intervention would reduce postprandial endotoxemia in association with reduced intestinal permeability. Data in Figure 4A demonstrate that postprandial endotoxin concentrations exhibited a significant time × treatment interaction (*p* = 0.004). Of interest, time-matched responses at 90 min were significantly lower in DGA + POTATO compared with DGA + BAGEL. The only other observed difference was higher endotoxin concentrations at 90 min in DGA + BAGEL compared with concentrations at 60 min in DGA + POTATO. In agreement with these effects, between-trial endotoxin AUC_0–120 min_ indicated overall lower responses in DGA + POTATO compared with DGA + BAGEL (*p* = 0.044; Figure 4B). In our examination of gut permeability based on urinary excretion of non-digestible sugars, we observed that lactulose/mannitol (0–5 h) and sucralose/erythritol (0–5 h and 6–24 h) did not differ between study arms (Figure 4C). However, lactulose/mannitol (6–24 h) was lower in DGA + POTATO compared with DGA + BAGEL (*p* = 0.03) in agreement with higher endotoxin levels in DGA + BAGEL (Figure 4B).

### 3.7. Intervention Effects on Microbiome Composition and Function

Based on a 7-point Bristol stool scale, the consistency of stool samples did not differ between DGA + BAGEL and DGA + POTATO (3.8 ± 0.22 vs. 4.0 ± 0.34, *p* = 0.46). In our LC/MS-based examination of SCFAs (Table 5), the fecal abundance of total SCFAs tended to be ~14% lower in DGA + POTATO compared to DGA + BAGEL (*p* = 0.09). Data were therefore transformed to percent abundance for each SCFA to improve interpretation. Although the proportions of branched-chain SCFAs did not differ between treatment arms (*p* > 0.05; Table 5), the proportion of acetate was increased in DGA + POTATO whereas propionate and valerate were lower compared with DGA + BAGEL.

**Figure 5:**
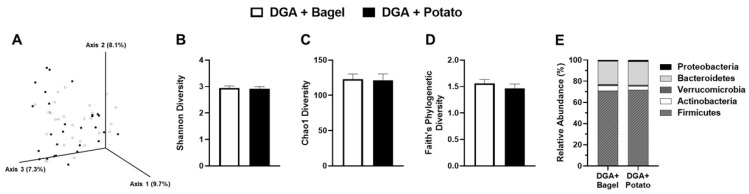
Gut microbial diversity and relative abundance in fecal samples collected from MetS persons who followed a *Dietary Guidelines for Americans*-based diet containing potatoes (DGA + POTATO) or bagels (DGA + BAGEL) for 2 w. (**A**) Principal component analysis of Bray-Curtis dissimilarity (β-diversity), and (**B**) Shannon, (**C**) Chao1, and (**D**) Faith’s Phylogenetic Diversity indexes of α-diversity were determined. (**E**) Relative abundance of phylum level bacteria populations. Group separation (β-Diversity) was tested by permutational multivariate analysis of variance (PERMANOVA). No statistically significant between-treatment effects were observed for indices of α-diversity or the differential abundance at the phylum level (*p* > 0.05).

To understand if the differences in SCFAs were attributed to changes in gut community structure, we also assessed microbiota composition. Principal component analysis showed that between-treatment microbiome composition had no visible treatment-wise separation, which was confirmed statistically (P_PERMANOVA_ > 0.05; Figure 5A). There were no significant differences between DGA + BAGEL vs. DGA + POTATO for measures of α-diversity including Shannon diversity index; Chao1 species richness; and Faith’s phylogenic diversity (*p* > 0.05; Figure 5B–D). Phylogenic bacteria community structure was unaffected between treatment arms at the phylum level (Figure 5E). Differential abundance analysis conducted using ANCOM II, which accounts for repeated measures while applying high power and a low false discovery rate threshold [47], identified only two between-treatment effects. Specifically, *Lachnospiraceae ND3007* group (+22%) and *Ruminococcaceae UCG*-005 (+42%) were enriched to a greater extent in DGA + POTATO compared with DGA + BAGEL.

## 4. Discussion

Findings of this randomized controlled trial demonstrate that compliance to a eucaloric *DGA*-based diet can improve select aspects of cardiometabolic health in MetS persons, especially glycemia, insulin resistance, and resting blood pressure. Although we hypothesized that daily potato consumption would enhance any observed benefits mediated by a *DGA* dietary pattern, our findings indicated that VEF (primary outcome), nitric oxide homeostasis, and postprandial metabolic and lipid peroxidation responses were unaffected by potatoes following an oral glucose challenge. These neutral findings contradict some observational studies suggesting that potato consumption increases cardiometabolic risk [10,11,12,13]. Consistent with our hypothesis, novel evidence demonstrates that daily potato consumption as part of a *DGA* diet protects against postprandial endotoxemia that was otherwise induced by a glucose challenge, but without affecting fasting circulating endotoxin (secondary outcome). This was accompanied by reduced small intestinal but not colonic permeability and an increased fecal acetate proportion, but limited between-treatment differences in gut bacteria community structure. These outcomes, therefore, support a *DGA* diet to alleviate cardiometabolic risk, and that incorporation of potatoes into a healthy dietary pattern can enhance gut barrier function.

Most Americans have poor dietary quality and do not adhere to the *DGA* recommendations [9]. Further, their intakes of vegetables, including starchy vegetables such as potatoes, do not meet recommendations. Contradicting recommendations of the *DGA*, evidence from some observational studies suggests that higher potato intakes increase cardiometabolic risk [10,11,12,13,50]. Because such studies are often limited in assessing the method of food preparation or considering comprehensive dietary patterns, controlled trials are critical for advancing an understanding of how foods can influence human health towards a goal of establishing evidence-based dietary recommendations. In the present study, we controlled participants’ basal diets to achieve high adherence to a *DGA* dietary pattern. We also circumvented a diet quality concern surrounding how potatoes are most commonly consumed (e.g., chipped, and fried), which has been linked to cardiometabolic risk [50], by providing baked or mashed potatoes with minimal dietary energy added. Due to practical concerns of facilitating a high compliance-controlled feeding intervention, prescribed foods were prepared in bulk for portioning and off-site consumption. This approach required that potatoes be prepared and refrigerated, which increases resistant starch content [51]. We were therefore able to deliver 17.5 g/d of resistant starch compared with low resistant starch intakes of Americans (1.9 g/1000 kcal) [52]. While a prescribed diet is a strength of our intervention, the study was not designed to assess the independent benefits of potato resistant starch on health outcomes; future studies will be needed to consider this dietary component. Nonetheless, our approach yielded excellent dietary compliance based on >94% consumption of test foods along with near-maximal HEI scores that indicated exceptional adherence to the prescribed DGA diet. Importantly, participants remained weight stable during and between each intervention phase consistent with our objective to assess health outcomes independent of any change in body mass.

Findings of our acute intervention demonstrated that 2-week intervention to a DGA diet was sufficient to reduce fasting glucose, insulin, insulin resistance (HOMA-IR), and blood pressure. Although improvements in these outcomes were statistically significant, the observed changes were modest and insufficient to reclassify study participants, on average, as normoglycemic or not fulfilling MetS criteria. However, this permitted an ability to evaluate potatoes on gut health and postprandial metabolic health independent of complex physiological interactions that would preclude data interpretation. Nonetheless, acute improvements in fasting cardiometabolic health parameters support longer-term studies to fully evaluate the benefits of a *DGA* dietary pattern. This concept was considered in an 8-week controlled trial in overweight and obese women who were randomized to a prescribed *DGA* or a typical American diet [53]. The *DGA* diet lowered systolic blood pressure but did not affect glycemia or insulinemia, which potentially reflects the parallel design of this trial or the relatively lower adherence (88%) to the prescribed diets compared with our study. Another possibility is that our study, similar to this prior study [53], provided high dietary fiber (37–39 g/day), consistent with *DGA* recommendations. This dietary consideration, in the context of a cross-over design that would be expected to foster greater study power, may account for the observed improvements in cardiometabolic risk factors. Indeed, fiber intakes are poor among Americans, despite wide recognition that higher intakes are associated with a lower prevalence of cardiometabolic disorders [54]. However, we did not assess usual or pre-study energy or nutrient intakes among our participants. We, therefore, cannot ascertain the extent to which our prescribed diets improved overall diet quality. Future studies of longer duration should consider specific dietary components and/or HEI-dependent changes relative to cardiometabolic health outcomes.

Because metabolic excursions in the postprandial period are implicated in cardiometabolic risk [2], we hypothesized that DGA + POTATO compared with DGA + BAGEL would protect against glucose-induced impairments in vascular health. On the contrary, the daily inclusion of potatoes did not alleviate postprandial glycemia or insulinemia. Thus, consistent with our prior studies demonstrating that postprandial hyperglycemia transiently impairs VEF by increasing oxidative stress and decreasing nitric availability [5,34,55,56], we did not expect that these parameters would improve in DGA + POTATO. While others reported that a *DGA*-based diet in obese and overweight women did not affect fasting endothelial function [57], controlled studies are needed to evaluate the potential benefits of a *DGA*-based diet on postprandial VEF and nitric oxide homeostasis.

Metabolic endotoxemia has been reported to initiate obesity and insulin resistance in mice [58]. Hyperglycemia was also demonstrated to be causally responsible for driving intestinal permeability and the influx of microbial products to the systemic circulation [59]. This suggests that decreasing hyperglycemia itself, reducing populations of endotoxin-containing Gram-negative bacteria, and/or the translocation of gut-derived endotoxins could improve cardiometabolic health. Although no between-treatment differences in fasting endotoxin were observed in our study (Table 3), DGA + POTATO attenuated glucose-induced endotoxemia without affecting postprandial hyperglycemia. Our data also show that urinary lactulose/mannitol but not sucrose/erythritol was lower in DGA + POTATO. This suggests that the attenuation of postprandial endotoxin was at least partly attributed to decreases in small intestinal but not colonic permeability based on the known gut region-specific differences in absorption and microbial fermentation of these gut permeability probes [36,37]. We, therefore, examined fecal straight and branched SCFAs due to the recognition that these microbial-derived metabolites influence gut barrier integrity, immune function, and metabolic health [60]. Although most fecal SCFAs showed no between-treatment differences, the proportion of acetate was higher while that of propionate was lower in DGA + POTATO compared with DGA + BAGEL. The observed increase in acetate (mol%) may have contributed to restoring intestinal epithelial cell turnover [61] and/or improving intestinal defense against enteropathogenic bacteria [62] to limit endotoxemia that was otherwise induced by a glucose challenge. Consistent with evidence in rodents that potato resistant starch exerts prebiotic activity to alleviate unfavorable shifts in bacteria populations [63,64], we considered that endotoxemia was limited by daily potato consumption due to shifts in microbial community structure. Our findings show that potato consumption had no effect on measures of diversity (i.e., evenness, and richness) and very limited effects on gut community structure. Our outcomes, however, are consistent with a recent crossover study in women indicating modest changes in the microbiome following 4-week consumption of a potato side dish (1 serving/day) compared with refined grain comparison foods [65]. Notably, fecal SCFAs (butyrate, acetate, and propionate) were unaffected, but butyrate-producing *Roseburia faecis* was increased following potato consumption [65]. It is possible that any prebiotic effect of potatoes in the present study could be masked by the substantial dietary modifications imposed by the DGA diet, but no fecal samples from the pre-intervention period were collected to make these comparisons. Further, our more limited observations on microbiota composition compared with this earlier report [65] may reflect that we controlled the entire basal diet rather than only potato test foods. Nonetheless, of the specific bacteria populations affected in DGA + POTATO, none were from the Gram-negative Proteobacteria phylum suggesting that potato-mediated decreases in postprandial endotoxemia were independent of altering the availability of endotoxin-containing populations. We did, however, observe increases in *Lachnospiraceae ND3007* [66] and *Ruminococcaceae (_UCG_005)* [67] that potentially contributed to a greater proportion of fecal acetate. Our evidence therefore suggests that the daily inclusion of potatoes, likely through their prebiotic resistant starch [19], enhanced acetate proportion to limit gut barrier dysfunction, and hyperglycemia-induced endotoxin translocation. Future work is needed to examine the independent effects of a *DGA*-based diet and potential dose-response effects of potatoes on microbiota composition and function.

In conclusion, resistant starch-containing potatoes protect against gut barrier dysfunction and glucose-stimulated endotoxemia in persons with MetS. That these benefits occurred in a background of a controlled *DGA*-based diet, which improved select cardiometabolic risk factors, suggests that an opportunity exists to modify the *DGA* dietary pattern to further enhance human health. While our findings support resistant starch-containing potatoes and a *DGA* diet, the acute duration of this controlled trial warrants future longer-term studies that can confirm these benefits. However, our initial findings support the concept that potatoes, at least when specifically prepared to enhance resistant starch content, can be effectively incorporated into a *DGA* dietary pattern without any adverse cardiometabolic health effects that are frequently observed in observational studies.

## Figures and Tables

**Table 1:** Energy and select nutrient content of potato and bagel.

Nutrient	Bagel (100 g, Plain)	Potato (350 g, Raw)
Energy (kcal) ^1^	275	275
Total Fat (g)	1.6	0.3
Protein (g)	10.5	7.47
Carbohydrate (g)	53.4	63.2
Total Dietary Fiber (g)	2.3	4.5
Resistant Starch (g) ^2^	0	17.5
Sodium (mg)	534	17
Potassium (mg)	101	1455

^1^ Data obtained from the United States Department of Agriculture, Agricultural Research Service, FoodData Central [28]. ^2^ Resistant starch content was determined using a commercially available spectrophotometric kit based on AOAC 2002.02 methodology (Megazyme, Wicklow, Ireland; #K-RSTAR); the reported mean value is derived from the analysis of a baked potato that was subsequently refrigerated for 24 h, 48 h, and 72 h (*n* = 3 replicates/time point) consistent with the prescribed 4-day dietary regimen of participants. No time-dependent differences in resistant starch content were observed between 24–72 h sample (*p* > 0.05), and resistant starch content at all time points was significantly greater (*p* < 0.05) than that of freshly baked potato (<1.9%, *w*/*w*).

**Table 2:** Characteristics of MetS persons who completed a randomized controlled trial that examined 2-w consumption of a *Dietary Guidelines for Americans*-based diet containing a daily serving of a potato or bagel.

	All (*n* = 27)	Male (*n* = 13)	Female (*n* = 14)	*p*
Age (year) ^1^	32.5 ± 1.3	31.9 ± 1.5	33.1 ± 2.1	0.67
BMI (kg/m^2^)	35.0 ± 1.0	34.9 ± 1.0	35.1 ± 1.7	0.91
Waist Circumference (cm)	109.8 ± 2.4	114.5 ± 2.1	105.4 ± 3.8	0.051
SBP (mmHg)	120.5 ± 1.6	125.2 ± 2.3	116.1 ± 1.6	0.004
DBP (mmHg)	82.9 ± 1.1	84.5 ± 1.3	81.4 ± 1.7	0.15
Left cIMT (mm)	0.61 ± 0.02	0.58 ± 0.03	0.64 ± 0.03	0.24
Right cIMT (mm)	0.60 ± 0.02	0.57 ± 0.03	0.63 ± 0.03	0.18
Plasma Triglyceride (mg/dL)	127.6 ± 15.5	171.2 ± 25.5	87.0 ± 10.3	0.008
Plasma HDL-C (mg/dL)	36.7 ± 1.4	34.7 ± 2.3	38.5 ± 1.6	0.19
Plasma Glucose (mg/dL)	103.7 ± 1.6	100.9 ± 2.7	106.4 ± 1.5	0.09
Plasma Insulin (µIU/mL)	19.9 ± 4.4	13.3 ± 1.1	26.5 ± 8.4	0.15
HOMA-IR	5.2 ± 1.2	3.3 ± 0.3	7.1 ± 2.4	0.14
Plasma Ascorbic Acid (µmol/L)	40.9 ± 4.2	41.7 ± 6.2	40.2 ± 5.9	0.87
Plasma Uric Acid (µmol/L)	355.2 ± 16.7	419.4 ± 13.2	300.2 ± 19.0	<0.001
MetS Criteria ^2,3^				
3 Risk Factors (%)	67	62	71	0.70
4 Risk Factors (%)	22	23	21	>0.99
5 Risk Factors (%)	11	15	7	0.60
Waist Circumference (%)	96	92	100	0.48
HDL-C (%)	89	85	93	0.60
Glucose (%)	85	69	100	0.04
Blood Pressure (%)	44	54	36	0.45
Triglyceride (%)	37	62	14	0.02

^1^ Data are means ± SE or the proportion of participants meeting specific MetS-related criteria. ^2^ Proportion of study participants who met established diagnostic criteria for MetS [24]. Between-gender comparisons were performed using Fisher’s exact test for categorical data and an unpaired Student’s *t*-test for nominal data. ^3^ Abbreviations: BMI, body mass index; cIMT, carotid artery intima media thickness; DBP, diastolic blood pressure; HDL-C, high-density lipoprotein-cholesterol; HOMA-IR, homeostatic model assessment of insulin resistance; MetS, metabolic syndrome; and SBP, systolic blood pressure.

**Table 3:** Clinical and biochemical parameters of MetS persons who consumed a *DGA*-based dietary pattern containing a daily serving of a bagel (DGA + BAGEL) or potato (DGA + POTATO) for 2-week ^1,2^.

	DGA + BAGEL		DGA + POTATO				
Parameter	Day 0	Day 14	Day 0	Day 14	P_Time_	P_Treatment_	P_Interaction_
BMI (kg/m^2^)	35.6 ± 1.2	35.1 ± 1.1	35.7 ± 1.2	35.2 ± 1.1	0.24	0.53	0.36
Waist Circumference (cm)	108.5 ± 2.2	107.8 ± 2.4	108.8 ± 2.2	107.5 ± 2.4	0.20	0.83	0.86
SBP (mmHg)	123.1 ± 1.9	113.6 ± 1.9	119.1 ± 2.2	114.6 ± 1.8	0.0001	0.18	0.06
DBP (mmHg)	81.5 ± 1.3	75.9 ± 1.7	81.9 ± 1.5	77.7 ± 1.4	0.001	0.20	0.40
Glucose (mg/dL)	104.4 ± 2.6	101.9 ± 2.4	109.5 ± 2.8	102.3 ± 2.6	0.04	0.14	0.16
Insulin (μIU/mL)	21.1 ± 5.5	14.2 ± 2.0	17.0 ± 2.0	14.9 ± 2.4	0.03	0.39	0.23
HOMA-IR	5.5 ± 1.5	3.6 ± 0.5	4.7 ± 0.6	3.8 ± 0.6	0.02	0.48	0.38
Cholecystokinin (pmol/L)	15.9 ± 6.0	14.3 ± 3.1	17.5 ± 5.1	14.2 ± 3.4	0.40	0.90	0.75
Malondialdehyde (μmol/L)	1.87 ± 0.1	1.78 ± 0.1	1.86 ± 0.1	1.93 ± 0.1	0.81	0.13	0.11
Endotoxin (EU/mL)	26.6 ± 2.0	21.2 ± 2.9	22.3 ± 2.1	23.3 ± 2.7	0.35	0.55	0.14
NO_x_ (μmol/L)	28.6 ± 5.3	29.8 ± 3.7	23.9 ± 1.8	30.1 ± 2.3	0.17	0.62	0.36
Arginine (μmol/L)	62.0 ± 2.6	66.2 ± 2.8	66.4 ± 3.3	65.7 ± 2.8	0.51	0.39	0.18
ADMA (nmol/L)	577.2 ± 22.0	570.5 ± 17.9	592.6 ± 22.7	567.7 ± 23.1	0.29	0.58	0.37
ADMA/Arginine (nmol/μmol)	9.60 ± 0.45	8.97 ± 0.43	9.33 ± 0.49	8.95 ± 0.47	0.12	0.66	0.64
SDMA (nmol/L)	529.6 ± 16.8	581.7 ± 28.8	567.4 ± 21.3	586.9 ± 26.4	0.04	0.21	0.33
SDMA/Arginine (nmol/μmol)	8.91 ± 0.45	9.22 ± 0.71	9.02 ± 0.52	9.36 ± 0.63	0.49	0.76	0.97
Homoarginine (μmol/L)	1.88 ± 0.1	2.08 ± 0.2	2.04 ± 0.2	2.11 ± 0.2	0.07	0.10	0.24
Homoarginine/Arginine (nmol/μmol)	30.7 ± 2.0	31.7 ± 2.1	32.2 ± 2.3	32.6 ± 2.4	0.34	0.51	0.78

^1^ Data (means ± SE, *n* = 27) were analyzed using repeated measures 2-way ANOVA ^2^ Abbreviations: DBP, diastolic blood pressure; *DGA*, *Dietary Guidelines for Americans*; BMI, body mass index; HOMA-IR, homeostatic model assessment of insulin resistance; HDL-C, high-density lipoprotein cholesterol; ADMA, asymmetric dimethylarginine; SDMA, symmetric dimethylarginine; NOx, nitric oxide metabolites (i.e., sum of nitrate and nitrite); and SBP, systolic blood pressure.

**Table 4:** AUC_0–120 min_ for cardiometabolic health in MetS persons who were provided a DGA-based diet containing a daily serving of bagel (DGA + BAGEL) or potato (DGA + POTATO) for 2 weeks prior to receiving an oral glucose tolerance test and assessing postprandial responses at 30 min intervals for 2 h ^1,2^.

Parameter	DGA + BAGEL	DGA + POTATO	*p* ^1^
Glucose (mg/dL × min)	2649 ± 499	2808 ± 565	0.73
Insulin (μIU/mL × min)	5940 ± 670	7086 ± 1372	0.29
Cholecystokinin (pmol/L × min)	−196 ± 266	−67 ± 344	0.80
Malondialdehyde (μmol/L × min)	57.2 ± 6.8	45.4 ± 7.2	0.08
FMD (% × min)	−259 ± 75	−198 ± 71	0.48
NOx (μmol/L × min)	−240 ± 88	−433 ± 48	0.11
ARG (μmol/L × min)	−826 ± 187	−833 ± 182	0.98
ADMA/ARG (nmol/μmol × min)	66 ± 35	95 ± 29	0.47
SDMA/ARG (nmol/μmol × min)	76 ± 37	134 ± 34	0.25
homoARG/ARG (nmol/μmol × min)	0.39 ± 0.18	0.45 ± 0.12	0.78

^1^ Data (means ± SE, *n* = 27) were analyzed using a paired Student’s test (2-tail). ^2^ Abbreviations: AUC, area under the curve; *DGA*, *Dietary Guidelines for Americans*; MetS, metabolic syndrome; FMD, brachial artery flow-mediated dilation; ARG, arginine; ADMA, asymmetric dimethylarginine; SDMA, symmetric dimethylarginine; and homoARG, homoarginine.

**Table 5:** Fecal SCFA profile of persons with MetS who consumed a *DGA*-based diet containing bagel or potato for 2 weeks ^1,2^.

	DGA + BAGEL	DGA + POTATO	
	μmol/g Feces		*p*-Value
Acetate	52.3 ± 4.7	46.6 ± 3.9	0.19
Propionate	23.1 ± 2.2	19.3 ± 2.1	0.03
Isobutyrate	2.7 ± 0.2	2.3 ± 0.2	0.13
Butyrate	21.3 ± 2.7	17.9 ± 2.6	0.16
2-Methylbutryrate	1.6 ± 0.2	1.4 ± 0.1	0.23
Isovalerate	1.8 ± 0.2	1.6 ± 0.2	0.16
Valerate	3.1 ± 0.3	2.4 ± 0.3	0.02
4-Methylvalerate	0.04 ± 0.01	0.03 ± 0.01	0.23
Hexanoate	1.0 ± 0.3	0.7 ± 0.2	0.38
Total SCFA	106.9 ± 9.5	92.2 ± 8.5	0.09
	mol % of total SCFA		
Acetate	49.3 ± 1.0	51.6 ± 1.0	0.03
Propionate	21.9 ± 0.8	20.7 ± 0.8	0.03
Isobutyrate	2.9 ± 0.2	2.8 ± 0.2	0.82
Butyrate	18.4 ± 1.0	17.5 ± 1.1	0.39
2-Methylbutryrate	1.7 ± 0.2	1.7 ± 0.2	0.99
Isovalerate	2.0 ± 0.2	2.0 ± 0.2	0.84
Valerate	3.0 ± 0.2	2.8 ± 0.2	0.02
4-Methylvalerate	0.03 ± 0.0	0.03 ± 0.0	0.54
Hexanoate	0.8 ± 0.2	0.8 ± 0.2	0.74
	mol % of straight SCFA		
Acetate	52.8 ± 1.0	55.3 ± 1.1	0.02
Propionate	23.5 ± 0.8	22.1 ± 0.8	0.04
Butyrate	19.6 ± 1.1	18.7 ± 1.1	0.36
Valerate	3.3 ± 0.3	3.0 ± 0.2	0.04
Hexanoate	0.9 ± 0.2	0.9 ± 0.2	0.76
	mol % of branched SCFA		
Isobutyrate	44.3 ± 0.6	44.3 ± 0.6	0.99
2-Methylbutryrate	25.1 ± 0.4	25.4 ± 0.5	0.55
Isovalerate	29.8 ± 0.4	29.7 ± 0.4	0.86
4-Methylvalerate	0.8 ± 0.3	0.6 ± 0.2	0.39

^1^ Data (means ± SE, *n* = 27) were analyzed using a paired Student’s *t*-test (2-sided) to assess between-treatment effects. ^2^ Abbreviations: *DGA*, *Dietary Guidelines for Americans*; MetS, metabolic syndrome; and SCFA, short-chain fatty acid.

## Data Availability

Data are available from the study authors upon reasonable request.

## Supplementary Materials

The following supporting information can be downloaded at: https://www.mdpi.com/article/10.3390/nu14081545/s1, Table S1: Diet composition and Healthy Eating Index scores of representative 2500 kcal diets that were provided to participants who were assigned to follow a DGA-based diet containing bagel or potato for 2-week.
