# Supplementary material for: Daily Inclusion of Resistant Starch-Containing Potatoes in a Dietary Guidelines for Americans Dietary Pattern Does Not Adversely Affect Cardiometabolic Risk or Intestinal Permeability in Adults with Metabolic Syndrome: A Randomized Controlled Trial
Source: Nutrients. 2022 Apr 8;14(8):1545. doi: 10.3390/nu14081545 (PMC9026745; doi:10.3390/nu14081545)
Supplement: Supplementary file 1 [file nutrients-14-01545-s001.zip › nutrients-1635890-supplementary.pdf]

Supplementary Table S1. Diet composition and Healthy Eating Index scores of representative 2500 kcal diets that were provided to participants who were assigned to follow a *DGA*-based diet containing bagel or potato for 2-wk.[1, 2]

Dietary Component	DGA + Bagel	DGA + Potato
Energy (kcal)	2466	2460
Fat (% energy)	31.4	31.2
Saturated Fat (% energy)	7.3	6.8
Cholesterol (mg)	293	288
Carbohydrates (% energy)	50.7	51.8
Protein (% energy)	17.9	17.0
Total Dietary Fiber (g)	36.6	39.4
Soluble Dietary Fiber (g)	10.0	10.1
Insoluble Dietary Fiber (g)	26.6	29.2
Vitamin D (µg)	7.6	7.8
Calcium (mg)	1059	974
Sodium (mg)	2309	1976
Potassium (mg)	3572	4631
Iron (mg)	23.1	22.4
**Food Groups**		
Whole Fruit (servings)	3.8	3.6
Total Vegetables (servings)	4.2	3.6
Whole Grains (servings)	5.1	6.1
Dairy and Dairy Alternatives (servings)	4.0	3.4
Total Protein Foods (servings)	10.8	10.3
Refined Grains (servings)	5.2	2.9
Healthy Eating Index Score	98.7	98.3

[1] Diets were evaluated using Nutrition Data System for Research software. Reported values (means ± SE) are the 4-day average of the rotating 2500 kcal prescribed diet.

[2] Abbreviations: *DGA*, Dietary Guidelines for Americans.
